# Editorial: Exploring Cancer Metabolic Reprogramming through Molecular Imaging

**DOI:** 10.3389/fonc.2017.00079

**Published:** 2017-04-26

**Authors:** Franca Podo, Zaver M. Bhujwalla, Egidio Iorio

**Affiliations:** ^1^Istituto Superiore di Sanità, Rome, Italy; ^2^The Johns Hopkins University School of Medicine, Baltimore, MD, USA

**Keywords:** editorial, energy metabolism, cancer metabolic reprogramming, molecular imaging, imaging methods

**Editorial on the Research Topic**

**Exploring Cancer Metabolic Reprogramming through Molecular Imaging**

## Background and Purpose of the Topic

Oncogene-driven reprogramming of energy metabolism has been added in 2011 to the list of general cancer hallmarks originally introduced by Hanahan and Weinberg to rationalize the complexities of neoplastic diseases (1, 2). However, a growing body of evidence points today to the more general vision of a wider cancer metabolic reprogramming not restricted to the deregulated cellular bioenergetics linked to aerobic glycolysis (Warburg effect), but encompassing a more complex network of concerted biochemical reactions. This wider metabolic network is responsible for the redirection of carbon and phosphorus fluxes through pathways involved in nucleotide, neutral lipid, and phospholipid biosynthesis, as well as in the oncogene-driven production of second messengers essential to cell growth and tumor invasiveness in a hostile tumor environment. Multiple efforts addressed to elucidate the key mechanisms of this more comprehensive metabolic rewiring recently led to the identification of novel signatures of malignancy, thus providing the grounds for improving cancer diagnosis and monitoring tumor response to therapy using advanced molecular imaging approaches. Among these, magnetic resonance spectroscopy (MRS) and magnetic resonance spectroscopic imaging, positron emission tomography (PET), functional MR imaging, and optical imaging technologies, combined with latest-generation cellular imaging approaches, currently offer powerful means to explore and monitor the effects of cancer metabolic reprogramming, a most versatile molecular machinery to counteract the effects of the microenvironment and eventually resist the attack of anticancer treatments. The progress of high-tech engineering and molecular imaging methods, combined with genomic, proteomic, and phosphor-proteomic approaches, are progressively improving the effectiveness of image-based clinical examinations and provide the basis to design and preclinically evaluate new targeted anticancer therapies.

**Scheme 1:**
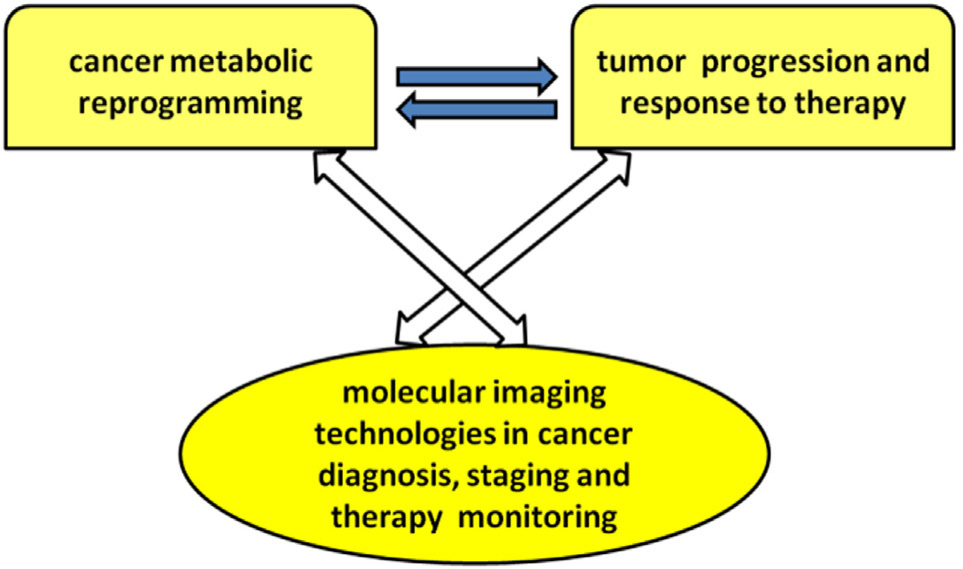
**Overview of research topic**.

The present research topic focuses on current achievements, challenges, and needs in the application of molecular imaging methods to explore different aspects of cancer metabolic reprogramming, with the final goal of improving individualized therapeutic decisions and patient outcome. Major attention has been focused on the links among oncogene-driven metabolic reprogramming, tumor progression, and response to therapy, as well as on the evolving capabilities of metabolic imaging technologies in cancer diagnosis, staging, and therapy monitoring (see Scheme 1).

The topic hosted 22 scientific contributions covering in a concerted manner these cutting-edge and cross-fertilizing research fields, including three general reviews, one opinion article, one perspective article, eight minireviews, and nine original articles, together with a combined list of over 90 keywords in oncology and metabolic imaging.

## Exploring the Links of Cancer Metabolic Reprogramming with Tumor Progression and Response to Therapy Using Molecular Imaging Approaches: Present Views and Perspectives

As noted in the opinion article by Serrao and Brindle, clinical oncology relies increasingly on biomedical imaging, with anatomical imaging, especially computed tomography (CT) and MRI, forming the mainstay of patient assessment, from diagnosis to treatment monitoring. However, the need for further improvements in specificity and sensitivity, coupled with imaging techniques that are reaching their limit of clinically attainable spatial resolution, has resulted in the emergence and growing use of imaging techniques with additional functional read-outs, such as 2-deoxy-2[^18^F]fluoro-d-glucose (^18^FDG)-PET and multiparametric MRI. These techniques add a new dimension to our understanding of the biological behavior of tumors, allowing a more personalized approach to patient management.

Compared to normal differentiated cells, cancer cells require a metabolic reprogramming to support their high proliferation rates and survival. A rewiring of energy metabolism through the Warburg effect is essential to generate the required biomass, including membrane biosynthesis, and to overcome bioenergetic and redox stress. Both established and evolving radioprobes developed in association with PET to detect tumor cell metabolism, and effects of treatment have been reviewed by Challapalli and Aboagye. In addition to providing us with opportunities for examining the complex regulation of reprogrammed energy metabolism in living subjects, the PET methods open up opportunities for monitoring pharmacological activity of new therapies that directly or indirectly inhibit tumor cell metabolism.

^1^H-MRS measurements have also been used to investigate tumor metabolism for diagnostic purposes. However, clinical applications of MRS have been hampered by low sensitivity and consequently low spatial and temporal resolution. Nuclear spin hyperpolarization of ^13^C-labeled substrates by dynamic nuclear polarization (DNP) radically increases the sensitivity of these substrates to detection by ^13^C MRS (3). DNP has rejuvenated interest in MRS measurements of tissue metabolism, as overviewed by Serrao and Brindle, with a focus on the potential clinical role for metabolic imaging with hyperpolarized [1-^13^C]pyruvate. Successful translation of this technique to the clinic was achieved recently with measurements of [1-^13^C]pyruvate metabolism in prostate cancer (4).

An original research article by Glickson and colleagues (Shestov et al.) on ^13^C MRS and LC-MS flux analysis of tumor intermediary metabolism presented the first validated metabolic network model for analysis of flux through key pathways of tumor intermediary metabolism including glycolysis, the oxidative and non-oxidative arms of the pentose pyrophosphate shunt, and the TCA cycle, as well as its anaplerotic pathways, pyruvate malate shuttling, glutaminolysis, and fatty acid biosynthesis and oxidation. Two models, respectively called bonded cumomer analysis for application to ^13^C MRS data and fragmented cumomer analysis for mass spectrometric data, are refined and efficient forms of isotopomer analysis that can be readily expanded to incorporate glycogen, phospholipid and other pathways, thereby encompassing all the key pathways of tumor intermediary metabolism. Results validated with melanoma and lymphoma cell models suggest the potential translation of these methods to *in situ* investigations on human cancer reprogramming using MRS with stable ^13^C isotopically labeled substrates on instruments operating at high magnetic fields (≥7 T), possibly in combination with FDG-PET and hyperpolarized ^13^C MRS methods.

Mutations in metabolic enzymes involved in cell bioenergetics but not directly responsible for aerobic glycolysis may also play an important role in cancer metabolic reprogramming. Notably, mutations in the metabolic enzyme isocitrate dehydrogenase (IDH), whose wild-type form catalyzes the interconversion of isocitrate to α-ketoglutarate, have recently been identified as drivers in the development of several tumor types. In particular, cytosolic IDH1 is mutated in 70–90% of low-grade gliomas and secondary glioblastomas, and mitochondrial IDH2 is mutated in about 20% of acute myeloid leukemia cases. An article by Ronen and colleagues (Viswanath et al.) provides a timely overview of the metabolic changes observed in mutant IDH cells and the various molecular imaging methods that have been used to characterize these changes. The review describes how metabolic imaging has helped shed light on the basic biology of mutant IDH cells and how this information can be leveraged to identify new therapeutic targets and develop new clinically translatable imaging methods to detect and monitor mutant IDH tumors *in vivo*.

As reviewed by Glunde and colleagues (Cheng et al.), recent evidence suggests that cancer cells undergo metabolic reprogramming beyond aerobic glycolysis and bioenergetic rewiring, in the course of tumor development and progression. Starting from pioneering studies at the end of the last century (5–7), a progressive awareness has been built on the impact of the MRS-detectable aberrant tumor phospholipid metabolism on oncogene-driven cell signaling perturbations, which lead to altered cell proliferation and block of cell differentiation (8, 9). In this context, all cancers tested so far display abnormal choline and ethanolamine phospholipid metabolism, which has been detected with numerous MRS approaches in cells, animal models of cancer, and the tumors of cancer patients. Since the discovery of this metabolic hallmark of cancer, many studies have been performed to elucidate the molecular origins of deregulated choline metabolism, to identify targets for cancer treatment, and to develop MRS approaches that detect choline and ethanolamine compounds for clinical use in diagnosis and treatment monitoring. Several enzymes in choline, recently also ethanolamine, and phospholipid metabolism [including choline kinase alpha (ChKα), phospholipase D1, phosphatidylcholine-specific phospholipase C (PC-PLC), sphingomyelinases, choline transporters, glycerophosphodiesterases, phosphatidylethanolamine *N*-methyltransferase, and ethanolamine kinase] have been shown to be involved in carcinogenesis and tumor progression, suggesting their potential use as targets for anticancer therapy, either alone or in combination with other chemotherapeutic approaches.

Besides aerobic glycolysis and altered choline metabolism, tumors are often characterized by peculiar microenvironment features such as hypoxia, vascular abnormalities, and low extracellular pH (pHe). The impact of these tumor characteristics has been investigated extensively in the context of tumor development, progression, and treatment response, resulting in a number of non-invasive imaging biomarkers. As highlighted by Ramamonjisoa and Ackerstaff, additional emerging evidence reveals that the interaction between tumor and stroma cells can alter tumor metabolism (leading to metabolic reprogramming) as well as tumor growth and vascular features. The review summarizes the current efforts to clarify how non-invasive multimodal imaging can help to characterize tumor–stroma interaction and understand its role in the development, progression, and treatment response of tumors.

The potential of induced metabolic bioluminescence imaging (imBI) to uncover metabolic effects of antiangiogenic therapy in tumors has been overviewed by Indraccolo and Mueller-Klieser. Tumor heterogeneity at the genetic level has been illustrated by a multitude of studies on the genomics of cancer, but whether tumors can be heterogeneous at the metabolic level is an issue that has been less systematically investigated so far. A burning related question is whether the metabolic features of tumors can change following either natural tumor progression (i.e., in primary tumors versus metastasis) or therapeutic interventions. In this regard, recent findings by independent teams indicate that antiangiogenic drugs cause metabolic perturbations in tumors, as well as metabolic adaptations associated with increased malignancy. ImBI is an imaging technique that enables detection of key metabolites associated with glycolysis, including lactate, glucose, pyruvate, and ATP in tumor sections. Signals captured by imBI can be used to visualize the topographic distribution of these metabolites and quantify their absolute amount. ImBI can be very useful for metabolic classification of tumors and to track metabolic changes in the glycolytic pathway associated with certain therapies. Imaging of the metabolic changes induced by antiangiogenic drugs in tumors by imBI or other emerging technologies is a valuable tool to uncover molecular sensors engaged by metabolic stress and offers an opportunity to understand how metabolism-based approaches could improve cancer therapy.

A perspective article by Podo and colleagues entitled “Activation of phosphatidylcholine-specific phospholipase C in breast and ovarian cancer: impact on MRS-detected choline metabolic profile and perspectives for targeted therapy” (Podo et al.) provides an overview of recent findings on functional and metabolic features of PC-PLC in breast and ovarian cancer cells in terms of (a) activation, protein overexpression, and subcellular redistribution of this enzyme in cancer cells compared with non-tumoral counterparts; (b) relative contributions of ChKα and PC-PLC to the intracellular MRS-detected phosphocholine pool; (c) interaction of PC-PLC with ErbB receptors’ family members such as human epidermal growth factor receptor 2 (HER2) and human epidermal growth factor receptor 1 (HER1, EGFR); and (d) effects of PC-PLC inhibition on HER2 overexpression, cell proliferation, and cell differentiation (10, 11). This body of evidence points to PC-PLC as a potential target for newly designed therapies, whose effects can be preclinically and clinically monitored by molecular imaging methods.

The unique capabilities of metabolic imaging to assess treatment response to cytotoxic and cytostatic agents were reviewed by Serkova and Eckhardt. For several decades, cytotoxic chemotherapeutic agents were considered the basis of anticancer treatment for patients with metastatic tumors. A decrease in tumor burden, assessed by volumetric CT and MRI, according to the Response Evaluation Criteria in Solid Tumors (RECIST), was considered as a radiological response to cytotoxic chemotherapies. In addition to RECIST-based dimensional measurements, a metabolic response to cytotoxic drugs can be assessed by PET using ^18^F-fluoro-thymidine (^18^FLT) as a radioactive tracer for drug-disrupted DNA synthesis. The decreased ^18^FLT-PET uptake is often seen concurrently with increased apparent diffusion coefficients by diffusion-weighted MRI (DWI) due to chemotherapy-induced changes in tumor cellularity. Recently, the discovery of molecular origins of tumorigenesis led to the introduction of novel signal transduction inhibitors (STIs). STIs are targeted cytostatic agents; their effect is based on a specific biological inhibition with no immediate cell death. As such, tumor size is no longer a sensitive end point for a treatment response to STIs; novel physiological imaging end points are needed. For receptor tyrosine kinase inhibitors, as well as modulators of the downstream signaling pathways, an almost immediate inhibition in glycolytic activity (the Warburg effect) and phospholipid turnover (the Kennedy pathway) has been seen by metabolic imaging in the first 24 h of treatment. The quantitative imaging end points by MRS and metabolic PET (including ^18^FDG and total choline) provide an early treatment response to targeted STIs, before a reduction in tumor burden can be seen.

## Challenges and Future Directions in the Application of Metabolic Imaging Approaches to Preclinical Models and to Cancer Patients

A series of 13 original articles or minireviews focused on MRI and metabolic imaging studies on cell-based models, dissected tissue specimens, and *in vivo* tissues especially in breast (Pais and Degani; Iorio et al.; Mori et al.; Haukaas et al.; Palma et al.; van der Kemp et al.; Sharma et al.; Sardanelli et al.), ovarian (Bagnoli et al.; Canese et al.; Penet et al.), and prostate cancers (Mori et al.; Selnaes et al.; Testa et al.).

The reported studies on breast cancer highlight relevant issues concerning development of quantitative molecular imaging methods that specifically detect estrogen receptor (ER) *in vivo* using novel ER-targeted contrast agents (Pais and Degani); identification of key players in choline metabolic reprogramming in triple negative breast cancer (Iorio et al.); influence of the tumor microenvironment on choline and lipid metabolism (Mori et al.); impact of freezing delay time on tissue samples for metabolomic studies (Haukaas et al.); monitoring of metabolic changes induced by gamma irradiation on breast tumor spheroids using ^1^H NMR spectroscopy and microimaging (Palma et al.); identification of ^31^P MRS phosphodiester signals of human fibroglandular breast tissue at ultrahigh field (van der Kemp et al.); and evaluation of the potential of DWI in the characterization of malignant, benign, and healthy breast tissues and molecular subtypes of breast cancer (Sharma et al.). An article entitled “Clinical breast MR using MRS or DWI: Who is the winner?” by Sardanelli et al. provides a critical summary of secondary evidence on two different *in vivo* non-contrast molecular imaging approaches, ^1^H MRS and DWI, with special focus on the translational perspective toward clinical feasibility and applicability.

Regarding epithelial ovarian carcinoma (EOC), a highly heterogeneous and lethal malignancy characterized by late diagnosis, frequent relapse, and development of chemoresistance, Bagnoli and colleagues reviewed the role of ChKα in sustaining the cancer “cholinic phenotype” (Bagnoli et al.). The article shows that ChKα inhibition, besides reducing ovarian cancer aggressiveness, increases disease sensitivity to drug treatment sparing normal cells and therefore opening a new therapeutic window. An abnormal tCho profile along with altered levels of other metabolites has also been detected in human EOC xenografts, as reviewed by Canese and colleagues (Canese et al.). This molecular imaging study provides a more extensive picture of tumor metabolism in EOC models *in vivo*, potentially opening the way to a multiple metabolic targeting. Furthermore, DWI is suggested as a potential tool for better differentiating malignant from benign tissues and possibly distinguishing cytotoxic from cytostatic treatment effects. New therapeutic strategies are urgently needed to improve survival of ovarian cancer patients. The effect of pantethine (precursor of vitamin B5 and active moiety of coenzyme A) on ovarian tumor progression and choline metabolism has been investigated by (Penet et al.) using MRI and high-resolution ^1^H MRS in a orthotopic ovarian cancer model. Pantethine treatment resulted in slower tumor progression, decreased levels of phosphocholine and phosphatidylcholine, and reduced metastases and ascites occurrence.

MRI can portray spatial variations in tumor heterogeneity, architecture, and tumor microenvironment, key biological features of prostate cancer. An original research article by Selnaes et al. focused on the relationships between MRI parameters measured on prostate cancer patients *in vivo*, individual metabolites measured *ex vivo* in prostatectomy specimens, and quantitative histopathology (Selnaes et al.). Last, but not least, Testa and colleagues reviewed the recent literature regarding molecular imaging methods developed and used to improve diagnosis and staging of prostate cancer (Testa et al.). The encouraging progress of *in vivo* metabolic imaging approaches nowadays points to the need of harmonized and shared protocols to increase the applicability of these technologies to a clinical setting. Furthermore, the use of well-characterized preclinical models that closely mirror the pathogenesis of human prostate cancer (e.g., the murine TRAMP model) allows for further progress in the comparative evaluation of DWI versus other molecular imaging approaches in assessing different stages of this disease (12).

## Author Contributions

All authors listed, have made substantial, direct and intellectual contribution to the work, and approved it for publication.

## Conflict of Interest Statement

The authors declare that the research was conducted in the absence of any commercial or financial relationships that could be construed as a potential conflict of interest. The reviewer, SB, declared a shared affiliation and a past coauthorship with one of the authors, ZB, to the handling editor, who ensured that the process nevertheless met the standards of a fair and objective review.
